# Association of inflammatory score with all-cause and cardiovascular mortality in patients with metabolic syndrome: NHANES longitudinal cohort study

**DOI:** 10.3389/fimmu.2024.1410871

**Published:** 2024-07-01

**Authors:** Yan Chen, Haonan Ju, Kailing Xie, Xin Zhao

**Affiliations:** ^1^ Department of Cardiology, Second Hospital of Dalian Medical University, Dalian, China; ^2^ Department of Second Clinical College, China Medical University, Shenyang, China

**Keywords:** inflammation, inflammatory score, metabolic syndrome, cardiovascular mortality, NHANES

## Abstract

**Background:**

Inflammatory scores are known to reflect the systemic inflammatory burden. Despite this, the association between the inflammatory score and the risk of all-cause and cardiovascular mortality in patients with metabolic syndrome (MetS) remains poorly understood. To address this gap in the literature, this study investigated this potential association between these two factors.

**Methods:**

A total of 3401 patients with MetS from the National Health and Nutrition Examination Survey (1999–2010) were enrolled. Survival status and cause of death were obtained by linking data from the National Death Index (NDI). The inflammatory score was calculated based on the sum of the Z-scores for white blood cell (WBC) count and C-reactive protein (CRP) at baseline. The patients were divided into inflammatory score quartiles. Cox proportional hazards regression was used to determine the association between inflammatory score and mortality. Restricted cubic splines (RCS) were used to explore the dose-response relationship between inflammatory score and mortality. Stratified analyses and interaction tests were conducted according to sex, age, body mass index (BMI), alcohol consumption, smoking status, hypertension, diabetes, and stroke status.

**Results:**

After a mean follow-up of 145.9 months, 1039 all-cause deaths and 295 cardiovascular deaths were recorded. The results of multivariate Cox regression analysis showed that compared to the lowest quartile (Q1), patients in the highest quartile (Q4) had a 1.74-fold increased risk of all-cause mortality (Model 3: HR = 1.74, 95%CI 1.30–2.32, *P* < 0.001) and a 1.87-fold increased risk of cardiovascular mortality (Model 3: HR = 1.87, 95%CI 1.12–3.13, *P* = 0.020). There was a ‘J’-shaped nonlinear relationship between the inflammatory score and all-cause mortality (*P* for nonlinearity = 0.001), and a marginally significant ‘J’-shaped relationship with cardiovascular mortality (*P* for nonlinearity = 0.057). The threshold points of the inflammatory score for adverse outcomes were - 0.643 and - 0.621, respectively.

**Conclusion:**

The inflammatory score is independently associated with increased all-cause and cardiovascular mortality in patients with MetS, and risk stratification of these patients using inflammatory scores may provide specific therapeutic strategies to improve their prognosis.

## Introduction

Metabolic syndrome (MetS), characterized by abdominal obesity, insulin resistance, abnormal blood pressure and lipid levels, has recently emerged as a major threat to global public health (1, 2). Analyzing NHANES data, Aguilar et al. found that the overall prevalence of metabolic syndrome in the United States was 33% (95%CI, 32.5%-33.5%) from 2003 to 2012 (3). In 2018, at least one billion people worldwide had MetS (4). The prevalence of MetS is known to increase with age (5). When combined with the current global aging trend, the global prevalence of MetS is expected to rise in coming years. According to a meta-analysis of a Southern African population, patients with MetS have a three-fold higher risk of stroke and cardiovascular diseases than individuals without MetS, and the possibility of dying from these diseases is twice that of individuals without MetS (6). Mottillo et al. found a significant association between metabolic syndrome and a 2-fold increased risk of cardiovascular events, as well as a 1.5-fold increase in all-cause mortality in a meta-analysis of 87 studies (7). In addition, MetS patients also have a significantly increased risk of premature death (8, 9). Therefore, MetS is a major cause of morbidity and mortality worldwide (4). In this context, it is necessary to accurately identify and screen patients with MetS who are at a high risk of death during clinical diagnosis and treatment to reduce the risk of adverse events.

Patients with MetS present with systemic low-grade inflammation (2, 10). This proinflammatory state can induce insulin resistance, and it is in this insulin-resistant state that the anti-inflammatory effect of insulin is disturbed, while also increasing the concentration of free fatty acids (FFAs), further exacerbating the inflammatory response (11, 12). Although previous studies have shown that white blood cell (WBC) count and C-reactive protein (CRP) is closely related to the risk of all-cause and cardiovascular mortality in patients with type 2 diabetes mellitus (T2DM) (13–16), the association between CRP levels and all-cause and cardiovascular mortality in patients with MetS or T2DM remains controversial (17). However, a reliable and stable comprehensive inflammatory index that reflects the overall inflammatory status of patients with MetS is lacking. Given that the actual number of patients with MetS is much larger than that of patients with diabetes (4) and that inflammation plays an important role in the progression in such patients (13), it is necessary to find a comprehensive index to assess the association between the inflammation and all-cause and cardiovascular mortality among MetS patients. The inflammatory score is currently considered an effective index that can provide an overall estimation of the inflammatory status (calculated based on the sum of the Z-scores of indicators, such as WBC and CRP) (18). Compared with individual inflammatory markers, the inflammatory score is more stable, comprehensive, and systematic in its ability to reflect the inflammatory burden of the body (18, 19). Several studies have confirmed the association between inflammatory scores and cardiovascular health, recurrent coronary events, and cancer risk (18–21), indicating that the inflammatory score may provide advantages when assessing the risk of adverse outcomes in the future. However, no studies have yet explored the relationship between the inflammatory score and the risk of all-cause and cardiovascular mortality in patients with MetS, which is significant for the daily care, treatment, and improvement of long-term prognosis in patients with MetS.

This study aimed to investigate the association between inflammatory score and all-cause and cardiovascular mortality in patients with MetS based on the National Health and Nutrition Examination Survey (NHANES) 1999–2010 and National Death Index (NDI) data.

## Methods

### Study population and data source

**Figure 1:**
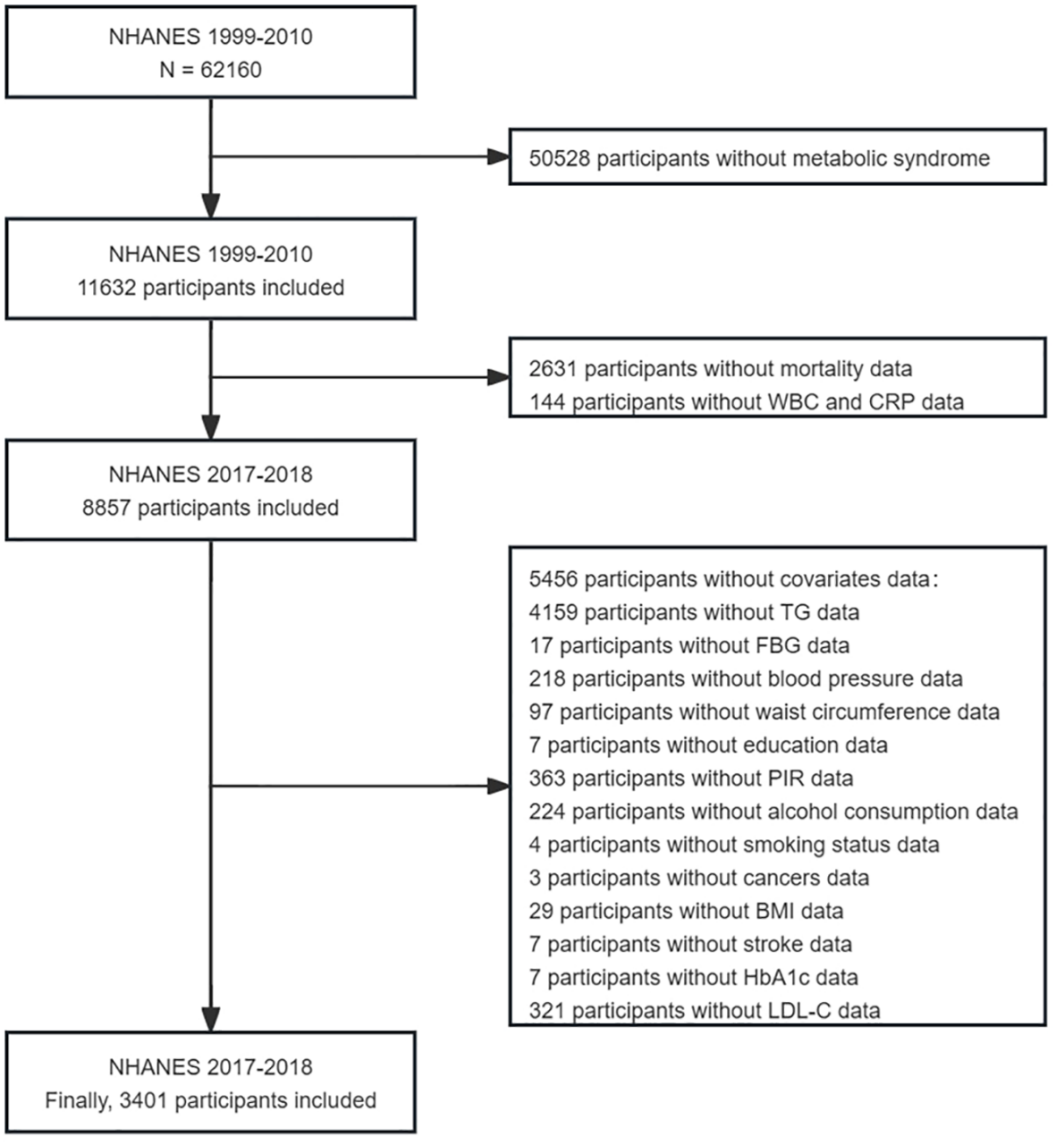
Flow chart of the sample selection from NHANES 1999–2010.

The NHANES, which began in the early 1960s and underwent a transformation in 1999, is a carefully planned study that comprehensively assesses the health and nutritional well-being (22). It has evolved into a continuous endeavor focusing on diverse health and nutrition metrics to cater to the evolving necessities. Each year, the survey meticulously examines a representative sample of approximately 5,000 individuals across the country. Data were gathered through face-to-face interviews and extensive health checks at mobile centers, employing a complex sampling method that ensured a representative cross-section of the population. Data for this study were taken from six cycles of the NHANES (1999–2010), involving 3401 eligible participants, all of whom were over 18 years old and were diagnosed with metabolic syndrome. Participants without complete survival or laboratory data were excluded (**Figure 1**).

MetS was diagnosed by the criteria set forth by the National Cholesterol Education Program’s Adult Treatment Panel III (NCEP-ATPIII) (23). Individuals meeting three or more of the following five conditions were considered to have metabolic abnormalities: (a) a waist circumference exceeding 102 cm for men or 88 cm for women; (b) triglycerides ≥ 1.7 mmol/L (150 mg/dL); (c) HDL-C < 1.03 mmol/L (40 mg/dL) for men, < 1.29 mmol/L (50 mg/dL) for women; (d) a systolic blood pressure (SBP) of 130 mmHg or higher, or a diastolic blood pressure (DBP) of 85 mmHg or higher; and (e) fasting blood glucose > 5.6 mmol/L (100 mg/dL) or above, or currently undergoing glucose-lowering treatment, or having a diagnosis of diabetes.

### Calculation of the inflammatory score

Z-scores were computed for each participant using their individual biomarker levels (X), the mean (M), and the standard deviation (SD) of the study population, according to the following formula: Z-score = (X − M)/SD. The inflammatory score was then calculated as the sum of the individual z-scores for CRP and WBC (18, 19).

### Study variables

The variables utilized in this study were extracted from four data sections of the NHANES: demographic, examination, laboratory, and questionnaire data. Demographic data included variables such as sex, age, race, education level, and income-poverty ratio (PIR). The examination and laboratory data predominantly included height, weight, waist circumference, blood pressure, HbA1c, fasting blood glucose, fasting TG, TC, LDL-C, HDL-C, WBC, and CRP levels. Height, weight, blood pressure, and waist circumference were measured at the Mobile Examination Center (MEC). All routine biochemical tests were performed in strict accordance with criteria specified in the NHANES Laboratory/Medical Technologist Manual of Procedures. Body mass index (BMI) was calculated as weight (kg) divided by the square of height (m). The smoking status was divided into never smokers, former smokers, and current smokers (24). Alcohol consumption is determined based on specific criteria: heavy drinking is defined as consuming ≥3 drinks per day for women, ≥4 drinks per day for men, or engaging in binge drinking on ≥5 days per month; moderate drinking is characterized by having two drinks per day for females, three drinks per day for males, or binge drinking on ≥2 days per month; mild drinking is designated for those who do not meet the criteria for heavy or moderate drinking, while never drinking refers to individuals who have consumed <12 drinks in their lifetime (25). Hypertension is diagnosed based on several factors, including a self-reported history of the condition, current use of antihypertensive medications, or having an average systolic blood pressure (SBP) ≥ 140 mmHg and/or an average diastolic blood pressure (DBP) ≥ 90 mmHg. The diagnosis of diabetes mellitus relies on several criteria: a self-reported physician diagnosis, fasting blood glucose (FBG) ≥ 7.0 mmol/L, HbA1c ≥ 6.5%, and/or the current use of diabetes medications. History of stroke or cancer was determined based on the results of a questionnaire survey.

### Assessment of survival status and cause of death

Survival data for all participants were obtained from the National Death Index (NDI) provided by the National Center for Health Statistics (NCHS), with data updated until December 31, 2019 (26). The participants’ baseline data were linked to survival data using the respondent sequence number (SEQN) in the NHANES to determine their survival outcomes and causes of death. The NCHS created the UCOD_113 variable to recode all deaths into comparable ICD-10-based cause of death groups. All-cause mortality was defined as death from any cause, including heart disease (codes 54–68), cancer (malignant neoplasms; codes 19–43), chronic lower respiratory disease (codes 82–86), unintentional injuries (codes 112–123), cerebrovascular diseases (code 70), Alzheimer’s disease (code 52), diabetes (code 46), pneumonia and influenza (codes 76–78), and influenza (codes 97–101). Cardiovascular death refers to death caused by heart disease. Additional information regarding survival data can be obtained from the following website: https://www.cdc.gov/nchs/data-linkage/mortality.htm.

### Statistical analysis

As recommended by the NHANES guidelines, we considered both complex sampling designs and sampling weights when analyzing NHANES data (27). The study participants were classified into four groups according to the quartiles (Q1-Q4) of their inflammatory scores. Basic characteristics were presented as counts and percentages (%) for categorical variables and medians (interquartile range, IQR) for continuous variables. The Chi-squared test was used for differences between groups for categorical variables, and the Kruskal-Wallis test was used for differences between groups for continuous variables. All-cause mortality and cardiovascular mortality were calculated during follow-up.

The probabilities of survival were calculated according to the Kaplan-Meier method and subsequently compared using Breslow and log-rank tests. Multivariate Cox proportional hazards models were used to evaluate the association between the inflammation score and all-cause and cardiovascular mortality in patients with MetS, and three models adjusted for different confounders were developed: Model 1: crude model; Model 2: adjusted for sex and age; Model 3: adjusted for sex, age, race, PIR, educational level, BMI, smoking status, alcohol consumption, hypertension, DM, cancer, stroke, and LDL-C level. To further clarify whether the association between inflammatory score and mortality was influenced by weight adjustment, we also performed sensitivity analyses for this association based on an unweighted Cox proportional hazards model, adjusting for the same confounders as in the weighted Cox regression analysis.

We used restricted cubic splines (RCS) to evaluate and visualize the dose-response relationship between the inflammatory score and mortality in the four knots. The number of knots was selected based on a combination of AIC values and clinical considerations. We estimated the threshold value and selected the inflection point with the highest likelihood. In addition, we conducted subgroup analyses based on sex (female, male) and age (<60 years, ≥60 years) to elucidate the patterns of the dose-response relationship between the inflammatory score and mortality across different sex and age subgroups.

Subgroup and interaction analyses were conducted to assess the influence of inflammatory score on all-cause and cardiovascular mortality. These analyses considered various stratifying factors, such as age (<60 years, ≥60 years), sex (female, male), BMI (<25 kg/m², 25–30 kg/m², ≥30 kg/m²), alcohol consumption (never, former, mild, moderate, heavy), smoking status (never, former, current), hypertension (no, yes), diabetes status (no, pre-diabetes, yes), and a history of stroke (no, yes). All statistical analyses were performed using the R (4.2.2) software. A *P*-value < 0.05 was considered statistically significant.

## Results

### Sample characteristics

A total of 3401 MetS patients with MetS were included and divided into four groups (Q1–Q4) according to the inflammatory score quartiles, as detailed in **Table 1**. The median age of the patients was 54 years, and 52.08% were female. Patients with higher inflammatory scores were more likely to be younger, female, current smokers, and have a higher BMI, waist circumference, and a greater number of risk factors for MetS (the number of MetS components) than those in the lowest quartile. In addition, significant between-group differences were observed in laboratory data, with patients with higher inflammatory scores having higher HbA1c, TG, CRP, and WBC counts than patients in the lowest quartile (all *P* < 0.05).

**Table 1:** Characteristic of participants.

Variables	Total (N=3401)	Q1(-2.26,-0.81)	Q2(-0.81,-0.28)	Q3(-0.28,0.46)	Q4(0.46,36.47)	P value
Age, (years)	54.00 (43.00,66.00)	56.00 (47.00,67.00)	56.00 (45.00,68.00)	54.00 (43.00,67.00)	50.00 (40.00,63.00)	< 0.0001
Sex,n (%)						< 0.0001
Female	1791 (52.08)	391 (44.04)	429 (50.01)	448 (53.51)	523 (61.03)	
Male	1610 (47.92)	459 (55.96)	422 (49.99)	401 (46.49)	328 (38.97)	
Race,n (%)						0.18
Non-Hispanic Black	548 (8.69)	162 (9.51)	126 (7.70)	114 (7.84)	146 (9.65)	
Mexican American	724 (7.38)	156 (5.93)	187 (7.40)	209 (8.68)	172 (7.61)	
Non-Hispanic White	1837 (76.60)	473 (78.96)	452 (76.39)	451 (75.68)	461 (75.27)	
Other Race	292 (7.32)	59 (5.61)	86 (8.51)	75 (7.80)	72 (7.46)	
Education levels,n (%)						0.01
< high school	1147 (21.58)	245 (17.55)	283 (21.10)	330 (26.46)	289 (21.49)	
= high school	894 (29.30)	222 (30.12)	229 (27.18)	216 (27.32)	227 (32.43)	
> high school	1360 (49.13)	383 (52.34)	339 (51.72)	303 (46.22)	335 (46.07)	
PIR,n (%)						< 0.0001
≤ 1	668 (12.63)	116 (8.36)	151 (10.11)	167 (12.22)	234 (19.91)	
1–3	1532 (39.29)	360 (35.10)	386 (40.64)	404 (40.11)	382 (41.50)	
> 3	1201 (48.08)	374 (56.54)	314 (49.25)	278 (47.68)	235 (38.59)	
Smoking status,n (%)						< 0.0001
Never	1624 (48.37)	474 (57.95)	424 (50.85)	368 (42.55)	358 (41.68)	
Former	1140 (32.67)	300 (34.50)	315 (35.49)	300 (34.23)	225 (26.52)	
Current	637 (18.95)	76 (7.55)	112 (13.66)	181 (23.23)	268 (31.80)	
Alcohol consumption,n (%)						0.06
Never	537 (13.08)	140 (13.89)	128 (11.57)	143 (14.20)	126 (12.66)	
Former	878 (23.50)	209 (22.51)	201 (21.13)	236 (26.14)	232 (24.29)	
Mild	1078 (34.50)	285 (36.65)	309 (40.61)	245 (31.48)	239 (29.19)	
Moderate	370 (12.95)	105 (14.06)	81 (11.57)	89 (11.84)	95 (14.22)	
Heavy	538 (15.97)	111 (12.89)	132 (15.12)	136 (16.33)	159 (19.63)	
BMI, kg/m²	31.52 (28.40,36.10)	30.19 (27.26,33.43)	31.09 (28.23,35.12)	32.30 (29.12,36.48)	33.89 (29.64,39.97)	< 0.0001
Waist circumference,cm	108.20 (100.10,118.00)	105.20 (98.20,112.90)	107.30 (99.20,117.00)	109.00 (101.80,119.00)	113.00 (103.00,123.50)	< 0.0001
SBP,mmHg	125.00 (115.00,138.00)	126.00 (115.00,138.00)	124.00 (114.00,137.00)	126.00 (115.00,137.00)	123.00 (113.00,138.00)	0.42
DBP,mmHg	72.00 (64.00,81.00)	74.00 (64.00,83.00)	72.00 (63.00,82.00)	72.00 (63.00,80.00)	72.00 (64.00,80.00)	0.04
DM,n (%)						0.23
No	1417 (44.54)	391 (48.66)	362 (45.79)	334 (43.02)	330 (40.51)	
Pre-DM	752 (23.82)	175 (22.54)	200 (25.00)	186 (23.46)	191 (24.31)	
Yes	1232 (31.65)	284 (28.81)	289 (29.20)	329 (33.51)	330 (35.18)	
Hypertension,n (%)						0.7
No	1054 (34.18)	262 (32.94)	253 (32.90)	266 (35.09)	273 (35.84)	
Yes	2347 (65.82)	588 (67.06)	598 (67.10)	583 (64.91)	578 (64.16)	
Stroke,n (%)						0.77
No	3186 (94.44)	802 (94.33)	807 (95.19)	790 (94.20)	787 (94.03)	
Yes	215 (5.56)	48 (5.67)	44 (4.81)	59 (5.80)	64 (5.97)	
Number of MetS components						<0.001
3 factors,n (%)	1978 (59.21)	566 (69.33)	496 (58.83)	450 (53.78)	466 (54.38)	
> 3 factors,n (%)	1423 (40.79)	284 (30.67)	355 (41.17)	399 (46.22)	385 (45.62)	
Laboratory data						
HbA1c,%	5.60 (5.40,6.10)	5.50 (5.30,5.90)	5.70 (5.40,6.00)	5.60 (5.30,6.10)	5.70 (5.40,6.20)	< 0.0001
FBG,mmol/L	5.94 (5.61,6.66)	5.94 (5.61,6.55)	5.94 (5.66,6.55)	5.94 (5.56,6.62)	5.98 (5.61,6.77)	0.48
TC,mmol/L	5.07 (4.37,5.84)	5.07 (4.27,5.77)	5.12 (4.45,5.95)	5.09 (4.40,5.82)	4.99 (4.32,5.79)	0.22
HDL-C,mmol/L	1.14 (0.98,1.32)	1.17 (0.98,1.40)	1.16 (0.98,1.37)	1.11 (0.98,1.29)	1.11 (0.93,1.27)	< 0.001
TG,mmol/L	1.89 (1.31,2.45)	1.76 (1.17,2.34)	1.92 (1.36,2.41)	2.00 (1.42,2.54)	1.92 (1.36,2.63)	< 0.0001
LDL-C,mmol/L	3.03 (2.38,3.65)	2.95 (2.35,3.60)	3.08 (2.43,3.70)	3.05 (2.33,3.65)	2.97 (2.35,3.60)	0.45
CRP,mg/dl	0.32 (0.14,0.65)	0.13 (0.07,0.23)	0.24 (0.14,0.40)	0.41 (0.22,0.67)	1.01 (0.51,1.62)	< 0.0001
WBC,10^9^/L	6.90 (5.80,8.20)	5.30 (4.70,5.70)	6.60 (6.20,7.10)	7.70 (7.10,8.30)	9.40 (8.10,10.60)	< 0.0001

Date are presented as median (IQR) or n (%).

PIR, poverty income ratio; BMI, body mass index; SBP, systolic blood pressure; DBP, diastolic blood pressure DM, diabetes mellitus; MetS, metabolic syndrome; FBG, fasting blood glucose; TC, total cholesterol; HDL-C, high-density lipoprotein cholesterol; TG, triglycerides; LDL-C, low-density lipoprotein cholesterol; CRP, c-reactive protein; WBC, white blood cell.

### Associations between inflammatory score and all-cause and cardiovascular mortality

**Figure 2:**
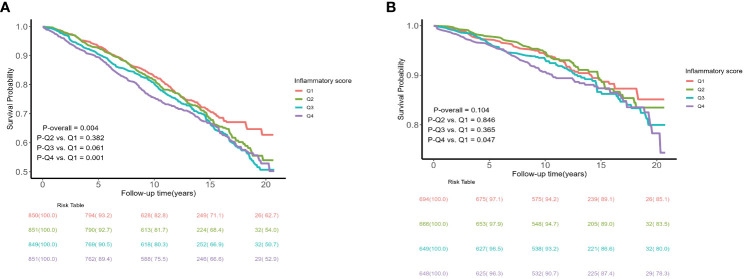
Kaplan–Meier curves of the survival rate and the number (%) of at-risk MetS patients with different levels of inflammatory score. **(A)** all-cause mortality; **(B)** cardiovascular mortality.

A total of 1095 all-cause and 295 cardiovascular deaths were recorded during the follow-up period. Kaplan-Meier survival plots indicated that the all-cause mortality and cardiovascular mortality of the population with an inflammatory score in the fourth quartile were both higher than those of the population in the first quartile (*P* < 0.05), as detailed in **Figure 2**. The comparison of differences between the different inflammatory score groups was based on the Breslow method and the results of the comparison of differences between groups based on the log-rank method are detailed in **Supplementary Figure 1**.

We constructed three Cox regression models to assess the association between the inflammatory score and all-cause and cardiovascular mortality in patients with MetS. Model 1 was a crude model; Model 2 was adjusted for sex and age factors; and Model 3 was adjusted for sex, age, race, PIR, educational level, BMI, smoking status, alcohol consumption, hypertension, DM, cancer, stroke, and LDL-C levels. Multivariate Cox regression analysis showed that compared to the lowest quartile (Q1), patients in the highest quartile (Q4) had a 1.74-fold increased risk of all-cause mortality (Model 3: HR = 1.74, 95%CI 1.30–2.32, *P* < 0.001) (*P* for trend < 0.001) and a 1.87-fold increased risk of cardiovascular mortality (Model 3: HR = 1.87, 95%CI 1.12–3.13, *P* = 0.02) (*P* for trend = 0.015), as detailed in **Table 2**. To further verify the robustness of this association, we employed an unweighted Cox regression analysis for sensitivity analysis. The results showed that unweighted Cox regression analysis was consistent with weighted Cox regression analysis in trends, and both showed a significant association between inflammatory score and all-cause and cardiovascular mortality in patients with MetS (all-cause mortality: Model 3: HR = 1.69, 95%CI 1.40–2.03, *P* < 0.0001; cardiovascular mortality: HR = 1.97, 95%CI 1.40–2.77, *P* < 0.0001) (*P* for trend < 0.001). This finding reinforces our conclusions, indicating that the observed association was robust and unaffected by weight adjustment, as detailed in **Supplementary Table 1**.

**Table 2:** Association between the inflammatory score and mortality in patients with metabolic syndrome.

Quantiles of the inflammatory score					
	Q1	Q2	Q3	Q4	*P* for trend
All-cause mortality					
Number of deaths	223	250	277	289	
Model 1 HR (95%CI) *P*-value	REF	1.10 (0.87–1.38)0.43	1.18 (0.91–1.52)0.22	1.31 (1.07–1.61)0.01	0.01
Model 2 HR (95%CI) *P*-value	REF	1.10 (0.88–1.38)0.39	1.33 (1.04–1.70)0.02	2.10 (1.65–2.69)<0.0001	<0.0001
Model 3 HR (95%CI) *P*-value	REF	1.08 (0.84–1.38)0.55	1.15 (0.87–1.51)0.33	1.74 (1.30–2.32)<0.001	<0.001
Cardiovascular mortality					
Number of deaths	67	65	77	86	
Model 1 HR (95%CI) *P*-value	REF	1.03 (0.71–1.49)0.88	1.33 (0.93–1.90)0.12	1.30 (0.89–1.90)0.18	0.08
Model 2 HR (95%CI) *P*-value	REF	1.13 (0.79–1.61)0.51	1.63 (1.10–2.42)0.01	2.51 (1.63–3.86)<0.0001	<0.0001
Model 3 HR (95%CI) *P*-value	REF	1.06 (0.72–1.56)0.77	1.31 (0.84–2.05)0.24	1.87 (1.12–3.13)0.02	0.015

Model 1: crude model;

Model 2: Adjusted for sex and age;

Model 3: Adjusted for sex, age, race, PIR, educational levels, BMI, smoking status, alcohol consumption, hypertension, DM, cancers, stroke, and LDL-C.

CI, Confidence Interval; REF, reference; PIR, poverty income ratio; BMI, body mass index; DM, diabetes mellitus; LDL-C, low-density lipoprotein cholesterol.

We also evaluated the association between CRP levels, WBC count, all-cause mortality, and cardiovascular mortality in patients with MetS. The results indicate that in MetS patients, CRP and WBC are closely associated with all-cause mortality (CRP: HR = 1.81, 95%CI 1.41–2.31, *P* < 0.0001; WBC: HR = 1.47, 95%CI 1.12–1.94, *P* = 0.01). However, the associations between CRP level, WBC count, and cardiovascular mortality were not significant (all *P* > 0.05), as shown in **Supplementary Tables 2**, **3**.

### Dose-response relationships between inflammatory score and all-cause and cardiovascular mortality

**Figure 3:**
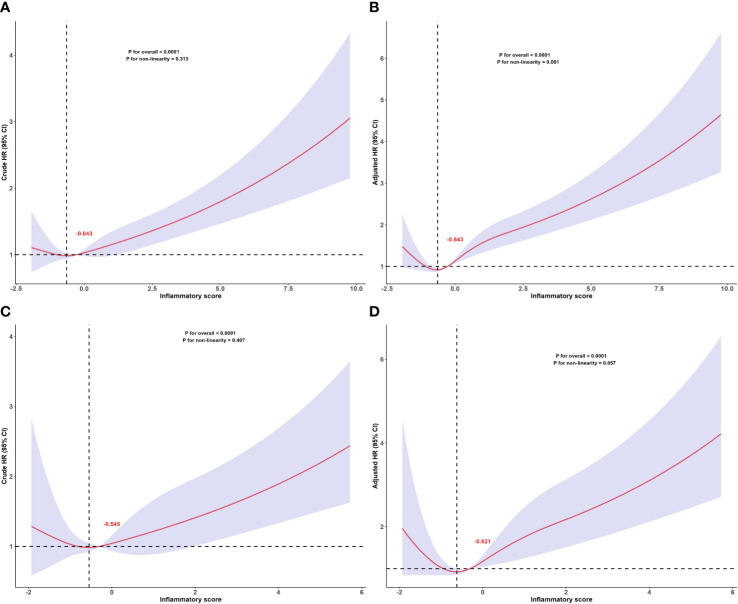
Dose-response relationship between inflammatory score and all-cause and cardiovascular mortality in patients with metabolic syndrome. Dose-response relationship between inflammatory score and all-cause mortality in patients with metabolic syndrome: **(A)** crude and **(B)** adjusted models. Dose-response relationship between inflammatory score and cardiovascular mortality in patients with metabolic syndrome: **(C)** crude and **(D)** adjusted models. The red numbers in the figure represent the inflammatory score corresponding to the threshold points. Adjusted for sex, age, race, PIR, educational levels, BMI, smoking status, alcohol status, hypertension, DM, cancers, stroke, LDL-C. The solid and shaded areas represent estimates and their corresponding 95% CIs, respectively (cardiovascular: cardiovascular disease; PIR, poverty income ratio; BMI, body mass index; DM, diabetes mellitus; LDL-C, low-density lipoprotein cholesterol).

The dose-response relationship between the inflammatory score and all-cause and cardiovascular mortality in patients with MetS was assessed based on the RCS, as shown in **Figure 3**. The RCS results showed a ‘J’-shaped nonlinear relationship between the inflammation score and all-cause mortality (*P* for non-linearity = 0.001) after adjusting for sex, age, race, PIR, educational level, BMI, smoking status, alcohol consumption, hypertension, DM, cancer, stroke, and LDL-C. A marginally significant ‘J’-shaped relationship was also observed with cardiovascular mortality (*P* for nonlinearity = 0.057). The threshold points of the inflammatory score for mortality were −0.643 (all-cause mortality) and −0.621 (cardiovascular mortality). When the inflammatory score exceeded −0.643, the risk of all-cause mortality increased with further elevation of the inflammatory score; similarly, when it surpassed −0.621, the risk of cardiovascular mortality also increased as the inflammatory score continued to increase.

**Figure 4:**
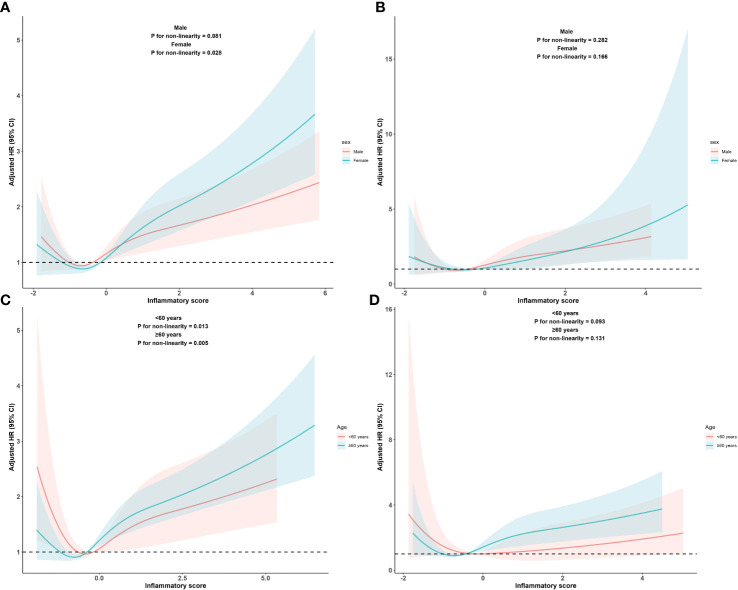
Dose-response relationship between inflammatory score and all-cause and cardiovascular mortality in metabolic syndrome patients within sex subgroups and age subgroups Dose-response relationship between inflammatory score and all-cause **(A)** and cardiovascular mortality **(B)** in metabolic syndrome patients within sex subgroups. Dose-response between inflammatory score and all-cause **(C)** and cardiovascular mortality **(D)** in metabolic syndrome patients within age subgroups. Adjusted for sex, age, race, PIR, educational levels, BMI, smoking status, alcohol status, hypertension, DM, cancers, stroke, LDL-C. The solid and shaded areas represent estimates and their corresponding 95% CIs, respectively (cardiovascular: cardiovascular disease; PIR, poverty income ratio; BMI, body mass index; DM, diabetes mellitus; LDL-C, low-density lipoprotein cholesterol).

In addition, we assessed the relationship between the inflammatory score and all-cause and cardiovascular mortality separately in the different age and sex subgroups. The RCS subgroup analysis results showed that the dose-response relationship between the inflammatory score and all-cause mortality, as well as cardiovascular mortality, remained consistent with the overall population trend across different age and sex subgroups, as shown in **Figure 4**.

### Subgroup analysis

Subgroup analysis was conducted to evaluate the association between the inflammatory score and mortality in different populations, as well as the presence of interaction effects. according to age (<60 years, ≥60 years), sex (female, male), BMI (<25 kg/m^2^, 25–30 kg/m^2^, ≥30 kg/m^2^), alcohol consumption (never, former, mild, moderate, heavy), smoking status (never, former, current), hypertension (no, yes), diabetes (no, pre-diabetes, yes), and stroke (no, yes). There was no significant interaction between the inflammatory score and the stratified variables (all *P* > 0.05), as detailed in **Tables 3**, **4**.

**Table 3:** Subgroup analysis of the association between inflammatory score and all-cause mortality.

All-cause mortality					
Inflammatory score	HR(95%CI)				*P* for interaction
	Q1	Q2	Q3	Q4	
Age, years					0.80
<60	REF	0.75 (0.36,1.57)	0.75 (0.41,1.37)	1.12 (0.62,2.02)	
≥60	REF	1.12 (0.89,1.39)	1.26 (0.96,1.65)	1.89 (1.40,2.56)	
Sex					0.66
female	REF	1.13 (0.80,1.58)	1.32 (0.87,2.00)	1.88 (1.23,2.87)	
male	REF	1.06 (0.79,1.41)	0.98 (0.71,1.36)	1.71 (1.21,2.42)	
BMI, kg/m^2^					0.53
<25	REF	1.83 (1.00,3.36)	1.77 (0.84,3.75)	4.07 (2.05,8.07)	
25–30	REF	1.16 (0.85,1.58)	1.31 (0.87,1.96)	2.45 (1.68,3.57)	
≥30	REF	0.98 (0.69,1.39)	0.93 (0.66,1.32)	1.32 (0.86,2.02)	
Alcohol consumption					0.42
never	REF	0.69 (0.41,1.19)	0.83 (0.49,1.41)	1.43 (0.83,2.46)	
former	REF	0.97 (0.60,1.57)	1.20 (0.74,1.96)	1.97 (1.20,3.23)	
mild	REF	1.33 (0.91,1.95)	1.25 (0.79,1.98)	1.43 (0.95,2.14)	
moderate	REF	1.31 (0.48,3.57)	1.28 (0.54,3.06)	5.47 (2.31,12.96)	
heavy	REF	1.01 (0.45,2.24)	0.82 (0.29,2.31)	1.36 (0.48,3.87)	
Smoking status					0.78
never	REF	0.94 (0.63,1.40)	1.24 (0.84,1.85)	1.97 (1.29,2.99)	
former	REF	1.00 (0.69,1.44)	1.16 (0.77,1.74)	1.70 (1.14,2.53)	
Current	REF	1.58 (0.64,3.89)	0.91 (0.43,1.92)	1.64 (0.77,3.51)	
Hypertension					0.18
no	REF	1.56 (0.77,3.14)	1.77 (0.87,3.57)	2.55 (1.39,4.69)	
yes	REF	0.98 (0.78,1.23)	1.02 (0.76,1.36)	1.61 (1.18,2.19)	
Diabetes					0.35
no	REF	1.17 (0.75,1.83)	1.16 (0.70,1.95)	1.89 (1.25,2.86)	
pre-diabetes	REF	0.74 (0.44,1.23)	1.50 (0.91,2.47)	1.95 (1.16,3.28)	
yes	REF	1.18 (0.81,1.70)	1.02 (0.71,1.46)	1.56 (1.01,2.41)	
Stroke					0.48
no	REF	1.04 (0.79,1.36)	1.14 (0.84,1.53)	1.78 (1.33,2.38)	
yes	REF	1.26 (0.72,2.21)	1.10 (0.55,2.17)	1.80 (0.88,3.66)	

**Table 4:** Subgroup analysis of the association between inflammatory score and cardiovascular mortality.

Cardiovascular mortality					
Inflammatory score	HR(95%CI)				*P* for interaction
	Q1	Q2	Q3	Q4	
Age, years					0.53
<60	REF	0.75 (0.23,2.42)	0.57 (0.19,1.76)	0.77 (0.24,2.50)	
≥60	REF	0.93 (0.62,1.39)	1.49 (0.92,2.41)	2.18 (1.26,3.76)	
Sex					0.18
female	REF	1.14 (0.60,2.20)	1.64 (0.74,3.65)	1.49 (0.67,3.36)	
male	REF	0.92 (0.46,1.84)	0.99 (0.60,1.64)	2.29 (1.17,4.48)	
BMI, kg/m^2^					0.32
<25	REF	1.47 (0.28,7.63)	1.48 (0.42,5.21)	6.32 (1.72,23.20)	
25–30	REF	1.55 (0.88,2.74)	1.99 (0.83,4.76)	2.91 (1.29,6.58)	
≥30	REF	1.14 (0.66,2.00)	1.12 (0.61,2.05)	1.43 (0.61,3.33)	
Alcohol consumption					0.31
never	REF	0.43 (0.13,1.43)	1.22 (0.52,2.84)	1.13 (0.43,2.95)	
former	REF	1.12 (0.49,2.57)	1.07 (0.53,2.19)	1.26 (0.50,3.16)	
mild	REF	1.26 (0.48,3.31)	1.53 (0.66,3.55)	2.42 (1.14,5.15)	
moderate	REF	0.16 (0.07,0.37)	0.24 (0.08,0.76)	9.10 (4.18,19.83)	
heavy	REF	5.20 (0.46,58.77)	1.56 (0.09,26.13)	2.45 (0.22,27.21)	
Smoking status					0.39
never	REF	1.04 (0.47,2.32)	1.78 (0.91,3.50)	1.88 (0.83,4.26)	
former	REF	0.61 (0.33,1.10)	1.03 (0.58,1.82)	1.82 (0.88,3.76)	
Current	REF	0.52 (0.08,3.20)	0.43 (0.08,2.42)	0.65 (0.16,2.65)	
Hypertension					0.23
no	REF	1.11 (0.27,4.55)	2.24 (0.71,7.06)	3.90 (1.06,14.35)	
yes	REF	0.97 (0.66,1.44)	1.13 (0.72,1.76)	1.63 (0.96,2.78)	
Diabetes					0.93
no	REF	0.88 (0.42,1.85)	0.93 (0.47,1.86)	1.76 (0.85,3.62)	
pre-diabetes	REF	1.25 (0.55,2.86)	2.97 (1.07,8.24)	2.55 (1.27,5.11)	
yes	REF	1.16 (0.58,2.33)	1.21 (0.61,2.40)	1.65 (0.70,3.89)	
Stroke					0.74
no	REF	1.08 (0.63,1.84)	1.42 (0.84,2.38)	2.21 (1.34,3.65)	
yes	REF	0.71 (0.21,2.39)	0.42 (0.11,1.63)	0.43 (0.07,2.75)	

**Supplementary Tables 4**, **5** present a subgroup analysis of the association between the inflammatory score and mortality without adjusting for potential confounders.

## Discussion

In this longitudinal cohort study based on NHANES 1999–2010 and NRI data, we found that inflammatory scores were significantly associated with all-cause and cardiovascular mortality in patients with MetS. In addition, the results based on RCS analysis showed a nonlinear, ‘J’-shaped dose-response relationship between inflammatory score and mortality in MetS patients.

Previous studies have primarily focused on the association among inflammatory scores, cardiovascular health, and cancer risk (18–21). However, no study has explored the association between inflammatory scores and all-cause or cardiovascular mortality in patients with MetS. The inflammatory score is typically composed of WBC and CRP; although there may be variations in the inflammatory markers included in the calculation of the inflammatory score across different studies, CRP and WBC are considered essential components (18). While several previous studies have demonstrated a correlation between either WBC or CRP levels and the risk of all-cause mortality or cardiovascular event mortality (13, 28–30), these studies have not combined WBC and CRP levels, which limits the objective evaluation of the association between inflammation and the risks of all-cause mortality and adverse cardiovascular events. In addition, previous studies have confirmed that combined inflammatory indicators based on leukocyte subtypes, such as the systemic immune inflammation index (SII, neutrophils × platelets/lymphocytes) and system inflammation response index (SIRI, neutrophils × monocytes/lymphocytes), have some value in predicting poor prognosis of all-cause or cardiovascular mortality (31–33). Although these indices are simple to calculate, they are limited in their ability to reflect the overall burden of body inflammation and do not avoid the impact of differences between individuals on the indices. Most importantly, these composite inflammatory indices usually do not include CRP, which is an important indicator for assessing the level of body inflammation (34). The inflammatory score was the sum of the Z-scores of the two most important inflammatory indicators in the body. Standardized processing of WBC and CRP can not only eliminate the effect of unit and magnitude differences on the score, making the score more accurate and objective, but also considers the effect of differences between individuals (18, 19, 21). The inflammation score cleverly combines two important indicators (CRP and WBC) reflecting the inflammatory status of the body to reflect the inflammatory burden of the body more comprehensively and also quantifies the current inflammatory level of the body, which has greater clinical application value than single inflammatory indexes.

The association between WBC or CRP and all-cause mortality and cardiovascular adverse events has been reported in patients with T2DM or MetS (13, 28, 30). A prospective cohort study of 7588 patients with recently diagnosed T2DM and no history of cardiovascular events by Gedebjerg et al. found that CRP levels were more strongly associated with the risk of all-cause mortality than with cardiovascular event mortality (30). Similarly, a large Danish cohort study based on the general population arrived at the same conclusion (15). Notably, Kuller found that WBC count, but not CRP level, was an independent predictor of death in men with MetS and coronary heart disease. Although these studies differed somewhat in study design, demographic characteristics, and analysis methods, they all found that the association between CRP level and cardiovascular events appeared to be significantly weaker than that with all-cause mortality in patients with T2DM or MetS. These findings contradict some known pathophysiological conclusions, namely the fact that CRP, a common inflammatory marker usually measured using immunoturbidimetry (serum), has been shown to be closely associated with cardiovascular and cerebrovascular diseases, such as sudden cardiac death, myocardial infarction, stroke, and peripheral arterial disease (16, 35, 36). CRP levels are also considered a reliable marker of low-grade inflammation (37). Previous studies have shown that MetS patients present a state of low-grade inflammation (2), and even in the early stages of common cardiovascular disease risk factors, such as overweight, prehypertension, and pre-diabetes, low-grade inflammation has been present in the body (38–40), and inflammation plays an important role in the occurrence, development, and poor prognosis of cardiovascular disease (41). Based on this evidence, the finding that the association between CRP levels and cardiovascular mortality is weaker or even non-significant compared with all-cause mortality observed in previous studies seems puzzling. Bruno et al. (28) found that the risk of all-cause and cardiovascular mortality within 5 years in T2DM patients with high CRP levels increased by 51% and 44% respectively, but there was a marginal significant between CRP and cardiovascular mortality (HR = 1.44, 95%CI 0.99–2.08). Considering that previous studies have suggested that the predictive power of CRP for adverse cardiovascular events may simply be due to its association with hyperglycemia, hypertension, and abnormal lipid metabolism, we adjusted for these factors in our final model. Our results suggest that compared to the lowest quartile, patients in the highest quartile had a 74% increased risk of all-cause mortality (HR = 1.74, 95%CI 1.30–2.32) and an 87% increased risk of cardiovascular mortality (HR = 1.87, 95%CI 1.12–3.13). This finding suggests that the inflammatory score can be used to effectively assess the risk of all-cause and cardiovascular mortality in patients with MetS. It is worth noting that in our study, the HR between the CRP level and all-cause mortality was higher than that between the inflammatory score and all-cause mortality. This discrepancy may be attributed to the fact that WBC somewhat weakens the association between the inflammatory score and all-cause mortality (42, 43), or the potential influence of confounding factors. Previous studies have confirmed the close association between inflammatory scores and adverse cardiovascular events (18, 19, 21, 44). Our findings also indicate that inflammatory scores possess a unique advantage in assessing the risk of cardiovascular mortality among patients with MetS. This may be due to the fact that patients with higher inflammatory scores tend to have a greater number of MetS components, such as obesity, insulin resistance, and elevated levels of blood pressure, blood lipids, and blood glucose. These risk factors collectively increase the risk of cardiovascular mortality among patients with MetS (13, 45–47).

It is worth mentioning that a ‘J’-type nonlinear association between inflammatory score and mortality was also found in our study, which suggests that the risk of all-cause and cardiovascular mortality increases with inflammatory score when the inflammatory score exceeds a certain level in MetS patients. Previous studies have confirmed that overweight and obese individuals have a systemic, low-grade inflammatory state (48, 49). In addition, CRP levels differed significantly among individuals of different genders (50). However, in our study, there was no interaction among the inflammatory score, BMI, and sex after adjusting for potential confounding factors, which seems to contradict previous research findings. The inflammatory state of the body is affected by many factors, such as diet, environment, disease, and stress. In addition, these results may be related to weighting factors, sample differences, study design, and genetic variability. However, these findings should be interpreted with caution. The underlying associations and mechanisms between the inflammatory score and all-cause and cardiovascular mortality in patients with MetS warrant further investigation.

### Strengths and limitations

This study has several limitations. First, in observational studies, it is difficult to confirm a causal association between the inflammatory score and mortality in patients with MetS. Secondly, in retrospective studies, it is difficult to avoid the influence of factors such as measurement outcomes and recall bias on the results. Furthermore, in this study, when participants were grouped according to their drinking consumption, the small number of participants in some subgroups may give rise to a sparse effect (51), which in turn could lead to an inflated or unstable HR. Next, the inflammatory score is sample-specific; therefore, the conclusions of this study may only apply to adult populations in the United States. In addition, due to the limitations of data completeness in the NHANES database, we were unable to incorporate additional inflammatory markers when constructing the inflammatory score. Moreover, the CRP and WBC counts were only measured at baseline, and dynamic data on CRP and WBC counts were not obtained. Relying solely on a single measurement of inflammatory factors may underestimate the true strength of the association.

Despite these limitations, our study is the first to reveal an association between the inflammation score and the risk of all-cause and cardiovascular mortality in patients with MetS, while clarifying the pattern of the dose-response relationship between the inflammation score and the risk of mortality in patients with MetS. This finding not only expands the scope and value of the inflammatory score in clinical practice but also emphasizes the importance of controlling the level of body inflammation to reduce the risk of death in patients with MetS. Most importantly, our study suggests that the inflammation score is a better choice for evaluating the risk of all-cause and cardiovascular mortality in patients with MetS, especially when evaluating the risk of cardiovascular mortality. Future studies should evaluate the association between inflammatory scores and mortality in different populations.

## Conclusions

Our study suggests that the inflammatory score is associated with all-cause and cardiovascular mortality in patients with MetS, with a particularly strong association with cardiovascular mortality. Additionally, we observed a nonlinear, ‘J’-shaped dose-response relationship between the inflammatory score and mortality in MetS patients. Based on these discoveries, clinicians may consider incorporating the inflammatory score into risk stratification strategies for patients with MetS to better define therapeutic strategies and improve prognosis.

## Data availability statement

The original contributions presented in the study are included in the article/**Supplementary Material**. Further inquiries can be directed to the corresponding author. All data regarding this study are available on the NHANES website (https://www.cdc.gov/nchs/nhanes/). This survey was approved by the National Center for Health Statistics Research Ethics Review Board. Informed consent was obtained from all participants.

## Ethics statement

The studies involving humans were approved by National Center for Health Statistics Research Ethics Review Board. The studies were conducted in accordance with the local legislation and institutional requirements. The participants provided their written informed consent to participate in this study.

## Author contributions

YC: Writing – review & editing, Writing – original draft, Validation, Methodology, Data curation, Conceptualization. HJ: Writing – original draft, Validation, Data curation, Conceptualization. KX: Writing – review & editing, Validation, Methodology. XZ: Writing – review & editing, Writing – original draft, Supervision, Resources.

## Supplementary material

Supplementary Figure 1 Kaplan–Meier curves of the survival rate and the number (%) of at-risk MetS patients with different levels of inflammatory score based on the log-rank test. **(A)** all-cause mortality; **(B)** cardiovascular mortality.
