# Supplementary material for: Association of inflammatory score with all-cause and cardiovascular mortality in patients with metabolic syndrome: NHANES longitudinal cohort study
Source: Front Immunol. 2024 Jul 1;15:1410871. doi: 10.3389/fimmu.2024.1410871 (PMC11246876; doi:10.3389/fimmu.2024.1410871)
Supplement: Supplementary file 1 [file Table_1.docx]

**Supplementary Table 1 Association between the inflammatory score and mortality in patients with metabolic syndrome (unweighted)**

| **Quantiles of the inflammatory score** | | | | | |
| --- | --- | --- | --- | --- | --- |
|  | **Q1** | **Q2** | **Q3** | **Q4** | ***P* for trend** |
| **All-cause mortality** |  |  |  |  |  |
| Number of deaths | 223 | 250 | 277 | 289 |  |
| Model 1 HR (95%CI) *P*-value | REF | 1.14(0.95-1.36)0.16 | 1.24(1.04-1.48)0.02 | 1.33(1.11-1.58)0.001 | <0.001 |
| Model 2 HR (95%CI) *P*-value | REF | 1.12(0.94-1.34)0.22 | 1.29(1.08-1.54)0.01 | 1.97(1.65-2.34)<0.0001 | <0.0001 |
| Model 3 HR (95%CI) *P*-value | REF | 1.10(0.91-1.32)0.32 | 1.16(0.97-1.40)0.10 | 1.69(1.40-2.03)<0.0001 | <0.0001 |
| **Cardiovascular mortality** |  |  |  |  |  |
| Number of deaths | 67 | 65 | 77 | 86 |  |
| Model 1 HR (95%CI) *P*-value | REF | 1.02(0.72-1.43)0.92 | 1.21(0.87-1.68)0.26 | 1.35(0.98-1.85)0.07 | 0.039 |
| Model 2 HR (95%CI) *P*-value | REF | 1.04(0.74-1.47)0.81 | 1.32(0.95-1.83)0.10 | 2.33(1.69-3.21)<0.0001 | <0.0001 |
| Model 3 HR (95%CI) *P*-value | REF | 1.00(0.70-1.41)0.98 | 1.13(0.81-1.59)0.47 | 1.97(1.40-2.77)<0.0001 | <0.001 |

Model 1: crude model;

Model 2: Adjusted for sex and age;

Model 3: Adjusted for sex, age, race, PIR, educational levels, BMI, smoking status, alcohol status, hypertension, DM, cancers, stroke, and LDL-C.

**Abbreviations:** CI, Confidence Interval; REF, reference; PIR, poverty income ratio; BMI, body mass index; DM, diabetes mellitus; LDL-C, low-density lipoprotein cholesterol.
