# Supplementary material for: Association of inflammatory score with all-cause and cardiovascular mortality in patients with metabolic syndrome: NHANES longitudinal cohort study
Source: Front Immunol. 2024 Jul 1;15:1410871. doi: 10.3389/fimmu.2024.1410871 (PMC11246876; doi:10.3389/fimmu.2024.1410871)
Supplement: Supplementary file 2 [file Table_2.docx]

**Supplementary Table 2 Association between the CRP and mortality in patients with metabolic syndrome**

| **Quantiles of the CRP** | | | | | |
| --- | --- | --- | --- | --- | --- |
|  | **Q1** | **Q2** | **Q3** | **Q4** | ***P* for trend** |
| **All-cause mortality** |  |  |  |  |  |
| Number of deaths | 241 | 254 | 261 | 283 |  |
| Model 1 HR (95%CI) *P*-value | REF | 1.10(0.85-1.43)0.46 | 1.11(0.84-1.46)0.46 | 1.24(0.99-1.55)0.06 | 0.079 |
| Model 2 HR (95%CI) *P*-value | REF | 1.16(0.93-1.45)0.19 | 1.35(1.05-1.73)0.02 | 2.06(1.61-2.64)<0.0001 | <0.0001 |
| Model 3 HR (95%CI) *P*-value | REF | 1.14(0.88-1.47)0.33 | 1.24(0.95-1.62)0.12 | 1.81(1.41-2.31)<0.0001 | <0.0001 |
| **Cardiovascular mortality** |  |  |  |  |  |
| Number of deaths | 77 | 68 | 68 | 82 |  |
| Model 1 HR (95%CI) *P*-value | REF | 0.90(0.58-1.38)0.62 | 1.11(0.76-1.62)0.60 | 1.02(0.66-1.55)0.94 | 0.704 |
| Model 2 HR (95%CI) *P*-value | REF | 1.01(0.67-1.53)0.95 | 1.44(0.96-2.17)0.08 | 2.07(1.33-3.23)0.001 | 0.001 |
| Model 3 HR (95%CI) *P*-value | REF | 0.92(0.59-1.43)0.70 | 1.20(0.77-1.88)0.42 | 1.61(0.98-2.63)0.06 | 0.065 |

Model 1: crude model;

Model 2: Adjusted for sex and age;

Model 3: Adjusted for sex, age, race, PIR, educational levels, BMI, smoking status, alcohol consumption, hypertension, DM, cancers, stroke, and LDL-C.

**Abbreviations:** CRP, C-reactive protein; CI, Confidence Interval; REF, reference; PIR, poverty income ratio; BMI, body mass index; DM, diabetes mellitus; LDL-C, low-density lipoprotein cholesterol.
