# Supplementary material for: Association of inflammatory score with all-cause and cardiovascular mortality in patients with metabolic syndrome: NHANES longitudinal cohort study
Source: Front Immunol. 2024 Jul 1;15:1410871. doi: 10.3389/fimmu.2024.1410871 (PMC11246876; doi:10.3389/fimmu.2024.1410871)
Supplement: Supplementary file 3 [file Table_3.docx]

**Supplementary Table 3 Association between the WBC and mortality in patients with metabolic syndrome**

| **Quantiles of the WBC** | | | | | |
| --- | --- | --- | --- | --- | --- |
|  | **Q1** | **Q2** | **Q3** | **Q4** | ***P* for trend** |
| **All-cause mortality** |  |  |  |  |  |
| Number of deaths | 238 | 269 | 250 | 282 |  |
| Model 1 HR (95%CI) *P*-value | REF | 1.06(0.81-1.39)0.68 | 0.89(0.70-1.12)0.32 | 1.30(1.05-1.62)0.02 | 0.06 |
| Model 2 HR (95%CI) *P*-value | REF | 0.97(0.75-1.25)0.82 | 0.98(0.75-1.27)0.86 | 1.83(1.44-2.31)<0.0001 | <0.0001 |
| Model 3 HR (95%CI) *P*-value | REF | 0.95(0.74-1.21)0.66 | 0.87(0.67-1.15)0.33 | 1.47(1.12-1.94)0.01 | 0.022 |
| **Cardiovascular mortality** |  |  |  |  |  |
| Number of deaths | 69 | 73 | 76 | 77 |  |
| Model 1 HR (95%CI) *P*-value | REF | 0.93(0.60-1.45)0.76 | 0.86(0.62-1.20)0.38 | 1.22(0.88-1.69)0.23 | 0.316 |
| Model 2 HR (95%CI) *P*-value | REF | 0.94(0.60-1.46)0.77 | 0.97(0.67-1.42)0.88 | 1.98(1.35-2.89)<0.001 | 0.002 |
| Model 3 HR (95%CI) *P*-value | REF | 0.86(0.54-1.36)0.51 | 0.85(0.59-1.23)0.39 | 1.47(0.97-2.23)0.07 | 0.101 |

Model 1: crude model;

Model 2: Adjusted for sex and age;

Model 3: Adjusted for sex, age, race, PIR, educational levels, BMI, smoking status, alcohol consumption, hypertension, DM, cancers, stroke, and LDL-C.

**Abbreviations:** WBC, white blood cell; CI, Confidence Interval; REF, reference; PIR, poverty income ratio; BMI, body mass index; DM, diabetes mellitus; LDL-C, low-density lipoprotein cholesterol.
