# Supplementary material for: Association of inflammatory score with all-cause and cardiovascular mortality in patients with metabolic syndrome: NHANES longitudinal cohort study
Source: Front Immunol. 2024 Jul 1;15:1410871. doi: 10.3389/fimmu.2024.1410871 (PMC11246876; doi:10.3389/fimmu.2024.1410871)
Supplement: Supplementary file 4 [file Table_4.docx]

**Supplementary Table 4 Subgroup analysis of the association between inflammatory score and all-cause mortality---Unadjusted for confounders**

| **All-cause mortality** | | | | | | |
| --- | --- | --- | --- | --- | --- | --- |
| **Inflammatory score** | **HR(95%CI)** | | | |  | ***P* for interaction** |
|  | **Q1** | **Q2** | **Q3** | **Q4** |  |  |
| **Age, years** |  |  |  |  |  | 0.44 |
| <60 | REF | 0.86(0.44,1.69) | 0.89(0.48,1.64) | 1.39(0.83,2.31) |  |  |
| ≥60 | REF | 1.25(0.98,1.59) | 1.37(1.09,1.73) | 1.99(1.58,2.51) |  |  |
| **Sex** |  |  |  |  |  | 0.02 |
| female | REF | 1.04(0.72,1.50) | 0.99(0.69,1.42) | 0.94(0.66,1.36) |  |  |
| male | REF | 1.13(0.89,1.44) | 1.38(1.00,1.92) | 1.92(1.43,2.58) |  |  |
| **BMI, kg/m^2^** |  |  |  |  |  | 0.08 |
| <25 | REF | 1.24(0.57,2.71) | 1.80(1.00,3.26) | 3.23(1.91,5.47) |  |  |
| 25-30 | REF | 1.39(1.02,1.89) | 1.63(1.10,2.40) | 1.89(1.36,2.63) |  |  |
| ≥30 | REF | 0.93(0.69,1.26) | 0.99(0.69,1.42) | 1.09(0.80,1.49) |  |  |
| **Alcohol consumption** |  |  |  |  |  | 0.59 |
| never | REF | 0.95(0.55,1.65) | 0.74(0.43,1.28) | 0.80(0.42,1.49) |  |  |
| former | REF | 0.98(0.65,1.49) | 1.35(0.89,2.05) | 1.60(1.09,2.34) |  |  |
| mild | REF | 1.23(0.86,1.75) | 1.22(0.84,1.76) | 1.31(0.88,1.95) |  |  |
| moderate | REF | 1.78(0.71,4.45) | 1.35(0.62,2.93) | 2.19(0.89,5.39) |  |  |
| heavy | REF | 0.94(0.40,2.17) | 0.87(0.33,2.29) | 1.07(0.43,2.69) |  |  |
| **Smoking status** |  |  |  |  |  | 0.74 |
| never | REF | 0.92(0.57,1.49) | 1.07(0.76,1.53) | 1.11(0.76,1.62) |  |  |
| former | REF | 1.21(0.90,1.62) | 1.41(0.97,2.04) | 1.59(1.17,2.17) |  |  |
| current | REF | 1.33(0.53,3.31) | 1.04(0.49,2.20) | 1.46(0.69,3.05) |  |  |
| **Hypertension** |  |  |  |  |  | 0.5 |
| no | REF | 1.22(0.61,2.45) | 1.73(0.96,3.11) | 1.71(0.98,2.98) |  |  |
| yes | REF | 1.06(0.83,1.34) | 1.09(0.83,1.43) | 1.28(1.06,1.54) |  |  |
| **Diabetes** |  |  |  |  |  | 0.28 |
| no | REF | 1.28(0.78,2.10) | 1.18(0.74,1.88) | 1.46(0.93,2.29) |  |  |
| pre-diabetes | REF | 0.75(0.50,1.12) | 1.29(0.86,1.93) | 1.13(0.72,1.76) |  |  |
| yes | REF | 1.14(0.74,1.77) | 0.97(0.67,1.41) | 1.11(0.77,1.58) |  |  |
| **Stroke** |  |  |  |  |  | 0.54 |
| no | REF | 1.06(0.81,1.38) | 1.12(0.84,1.50) | 1.28(1.02,1.61) |  |  |
| yes | REF | 1.72(0.80,3.71) | 1.73(0.90,3.31) | 1.66(0.75,3.65) |  |  |
