# Supplementary material for: Association of inflammatory score with all-cause and cardiovascular mortality in patients with metabolic syndrome: NHANES longitudinal cohort study
Source: Front Immunol. 2024 Jul 1;15:1410871. doi: 10.3389/fimmu.2024.1410871 (PMC11246876; doi:10.3389/fimmu.2024.1410871)
Supplement: Supplementary file 5 [file Table_5.docx]

**Supplementary Table 5 Subgroup analysis of the association between inflammatory score and cardiovascular mortality---Unadjusted for confounders**

| **Cardiovascular mortality** | | | | | | |
| --- | --- | --- | --- | --- | --- | --- |
| **Inflammatory score** | **HR(95%CI)** | | | |  | ***P* for interaction** |
|  | **Q1** | **Q2** | **Q3** | **Q4** |  |  |
| **Age, years** |  |  |  |  |  | 0.31 |
| <60 | REF | 1.15(0.50,2.64) | 0.78(0.25,2.48) | 1.28(0.44,3.68) |  |  |
| ≥60 | REF | 1.13(0.75,1.70) | 1.73(1.20,2.47) | 2.34(1.56,3.51) |  |  |
| **Sex** |  |  |  |  |  | 0.03 |
| female | REF | 1.19(0.60,2.37) | 1.25(0.65,2.41) | 0.73(0.38,1.39) |  |  |
| male | REF | 0.86(0.46,1.59) | 1.42(0.83,2.42) | 2.30(1.21,4.35) |  |  |
| **BMI, kg/m^2^** |  |  |  |  |  | 0.09 |
| <25 | REF | 0.46(0.11,1.94) | 1.84(0.65,5.17) | 3.25(1.25,8.46) |  |  |
| 25-30 | REF | 1.70(0.96,3.02) | 2.46(1.15,5.26) | 2.27(1.26,4.09) |  |  |
| ≥30 | REF | 0.99(0.64,1.52) | 1.06(0.66,1.71) | 1.04(0.58,1.86) |  |  |
| **Alcohol consumption** |  |  |  |  |  | 0.54 |
| never | REF | 0.46(0.16,1.35) | 1.08(0.50,2.34) | 0.57(0.27,1.20) |  |  |
| former | REF | 1.22(0.60,2.47) | 1.34(0.74,2.40) | 1.18(0.52,2.65) |  |  |
| mild | REF | 1.02(0.42,2.48) | 1.40(0.70,2.78) | 1.80(0.97,3.37) |  |  |
| moderate | REF | 0.90(0.19, 4.38) | 1.21(0.30, 4.93) | 3.10(0.89,10.85) |  |  |
| heavy | REF | 4.42(0.46,42.86) | 2.20(0.24,19.94) | 3.48(0.35,34.12) |  |  |
| **Smoking status** |  |  |  |  |  | 0.48 |
| never | REF | 1.12(0.51,2.44) | 1.49(0.82,2.71) | 1.12(0.56,2.28) |  |  |
| former | REF | 0.73(0.43,1.25) | 1.44(0.83,2.48) | 1.73(1.01,2.97) |  |  |
| current | REF | 1.67(0.42,6.69) | 0.86(0.24,3.17) | 1.13(0.35,3.70) |  |  |
| **Hypertension** |  |  |  |  |  | 0.53 |
| no | REF | 0.87(0.27,2.82) | 2.27(0.84,6.15) | 2.05(0.64,6.63) |  |  |
| yes | REF | 1.02(0.67,1.53) | 1.23(0.85,1.79) | 1.26(0.85,1.86) |  |  |
| **Diabetes** |  |  |  |  |  | 0.8 |
| no | REF | 0.87(0.43,1.79) | 1.32(0.72,2.42) | 1.39(0.63,3.10) |  |  |
| pre-diabetes | REF | 0.84(0.39,1.79) | 1.53(0.67,3.50) | 1.26(0.56,2.80) |  |  |
| yes | REF | 1.11(0.59,2.10) | 1.04(0.58,1.89) | 1.03(0.53,1.99) |  |  |
| **Stroke** |  |  |  |  |  | 0.88 |
| no | REF | 1.00(0.59,1.70) | 1.33(0.86,2.05) | 1.33(0.88,2.01) |  |  |
| yes | REF | 1.40(0.39,5.06) | 1.72(0.63,4.67) | 1.18(0.32,4.37) |  |  |
