# Supplementary figures and images for: Association of inflammatory score with all-cause and cardiovascular mortality in patients with metabolic syndrome: NHANES longitudinal cohort study
Source: Front Immunol. 2024 Jul 1;15:1410871. doi: 10.3389/fimmu.2024.1410871 (PMC11246876; doi:10.3389/fimmu.2024.1410871)

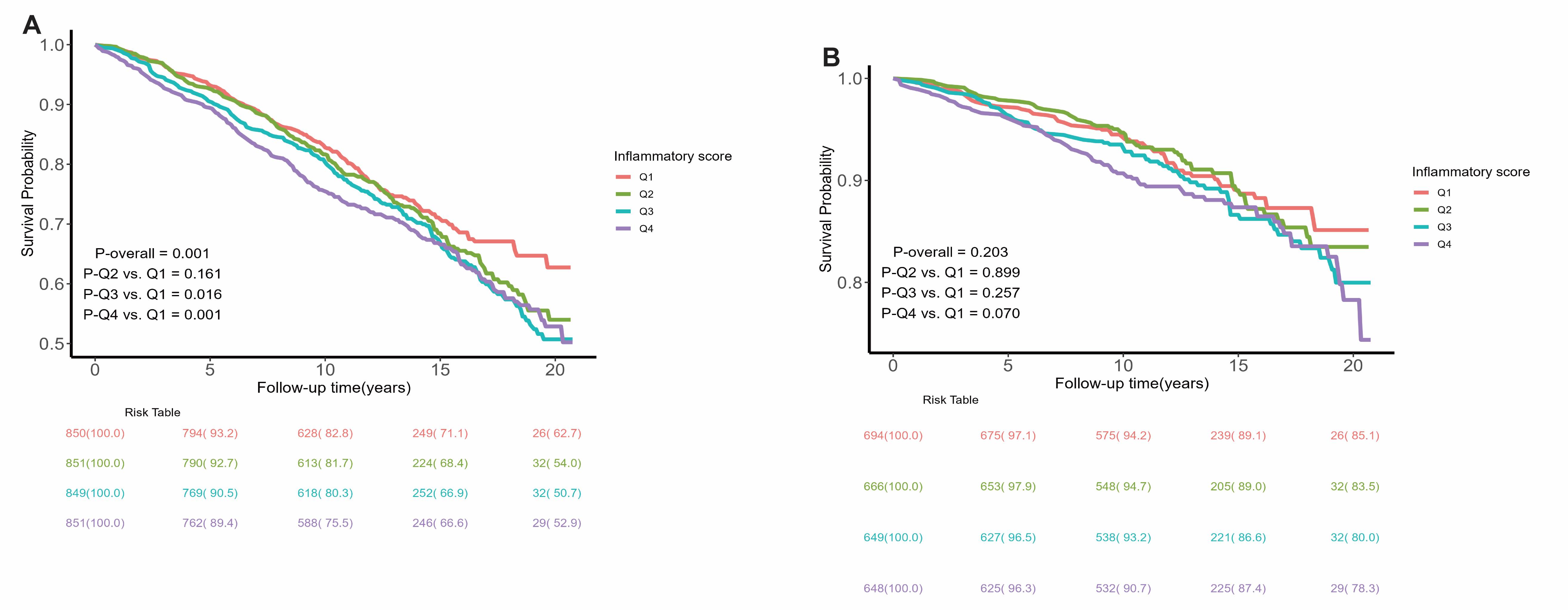

Supplement: Supplementary Figure 1 — Kaplan–Meier curves of the survival rate and the number (%) of at-risk MetS patients with different levels of inflammatory score based on the log-rank test. (A) all-cause mortality; (B) cardiovascular mortality. [file Image_1.jpg]
